# Fabrication and In Vitro Study of Tissue-Engineered Cartilage Scaffold Derived from Wharton's Jelly Extracellular Matrix

**DOI:** 10.1155/2017/5839071

**Published:** 2017-10-29

**Authors:** Tongguang Xiao, Weimin Guo, Mingxue Chen, Chunxiang Hao, Shuang Gao, Jingxiang Huang, Zhiguo Yuan, Yu Zhang, Mingjie Wang, Penghao Li, Jiang Peng, Aiyuan Wang, Yu Wang, Xiang Sui, Li Zhang, Wenjing Xu, Shibi Lu, Heyong Yin, Jianhua Yang, Shuyun Liu, Quanyi Guo

**Affiliations:** ^1^Institute of Orthopaedics, Chinese PLA General Hospital, Beijing Key Lab of Regenerative Medicine in Orthopaedics, Key Laboratory of Musculoskeletal Trauma & War Injuries, PLA, No. 28 Fuxing Road, Haidian District, Beijing 100853, China; ^2^First Department of Orthopaedics, First Affiliated Hospital of Jiamusi University, No. 348 Dexiang Road, Xiangyang District, Jiamusi 154003, China; ^3^Institute of Anesthesiology, Chinese PLA General Hospital, No. 28 Fuxing Road, Haidian District, Beijing 100853, China; ^4^Center for Biomaterial and Tissue Engineering, Academy for Advanced Interdisciplinary Studies, No. 5 Yiheyuan Road, Haidian District, Peking University, Beijing 100871, China

## Abstract

The scaffold is a key element in cartilage tissue engineering. The components of Wharton's jelly are similar to those of articular cartilage and it also contains some chondrogenic growth factors, such as insulin-like growth factor I and transforming growth factor-*β*. We fabricated a tissue-engineered cartilage scaffold derived from Wharton's jelly extracellular matrix (WJECM) and compared it with a scaffold derived from articular cartilage ECM (ACECM) using freeze-drying. The results demonstrated that both WJECM and ACECM scaffolds possessed favorable pore sizes and porosities; moreover, they showed good water uptake ratios and compressive moduli. Histological staining confirmed that the WJECM and ACECM scaffolds contained similar ECM. Moreover, both scaffolds showed good cellular adherence, bioactivity, and biocompatibility. MTT and DNA content assessments confirmed that the ACECM scaffold tended to be more beneficial for improving cell proliferation than the WJECM scaffold. However, RT-qPCR results demonstrated that the WJECM scaffold was more favorable to enhance cellular chondrogenesis than the ACECM scaffold, showing more collagen II and aggrecan mRNA expression. These results were confirmed indirectly by glycosaminoglycan and collagen content assessments and partially confirmed by histology and immunofluorescent staining. In conclusion, these results suggest that a WJECM scaffold may be favorable for future cartilage tissue engineering.

## 1. Introduction

Articular cartilage, especially knee and hip hyaline cartilage, plays an important role in bearing stress and lubricating the joint [1]. It is easily damaged by trauma or overuse; moreover, self-healing is difficult after any injury because of its avascular characteristics [2–4]. Damaged cartilage can lead to joint pain, swelling, and dysfunction, eventually causing degenerative arthritis with no effective therapeutic strategy available [5]. Although some approaches for ameliorating cartilage injuries have been attempted, these attempts often resulted in suboptimal outcomes. Three surgical techniques are currently used for repairing articular cartilage: microfracture (MF) repair, autologous chondrocyte implantation (ACI), and osteochondral autograft transfer (mosaicplasty) [6–8]. However, while the newly formed tissue is fibrocartilaginous in terms of MF and ACI, it lacks the native cartilage collagen network and is thus susceptible to failure [9–12]. Mosaicplasty is usually appropriate for cases with damage involving the subchondral bone. Unfortunately, there are limitations in obtaining sufficient autologous graft tissue to repair large-area cartilage injuries [13, 14].

Over the past two decades, there has been increasing interest in constructing a functional tissue-engineered cartilage as an alternative for cartilage regeneration [15, 16]. In particular, the use of scaffolds derived from decellularized cartilage extracellular matrix (ECM) to construct tissue-engineered cartilage has received a lot of attention [17–21]. Because decellularized cartilage ECM can preserve native bioactive proteins well, it provides a biomimetic microenvironment for cell attachment, proliferation, and redifferentiation [21]. Hyaline cartilage is composed primarily of the proteoglycan aggrecan and collagen type II [22].

The umbilical cord includes one vein, two arteries, and the surrounding myxomatous substance, which is referred to as Wharton's jelly; it contains substantial amounts of collagen, hyaluronic acid, and sulfated proteoglycans within its ECM components [23]. Wharton's jelly ECM is similar to cartilage ECM; however, it is also rich in peptide growth factors, including epidermal growth factor (EGF), platelet-derived growth factor (PDGF), a fibroblast growth factor (FGF), bFGF, insulin-like growth factor I (IGF-I), and transforming growth factor-*β* (TGF-*β*) [24, 25]. These peptide growth factors are conducive to the cellular biosynthesis of collagen and glycosaminoglycans (sGAG), especially IGF-I and TGF-*β* in chondrogenesis. Thus, Wharton's jelly ECM could be a good alternative biomaterial for tissue-engineered cartilage.

Given this background, we sought to compare the construction of tissue-engineered cartilage scaffolds using Wharton's jelly ECM (WJECM) with that using articular cartilage ECM (ACECM). We used a waterproof pulverization and differential centrifugation approach to obtain two different decellularized ECMs and prepared 3D scaffolds by a freeze-drying method. First, we compared the physicochemical properties between the two scaffolds. Then, we constructed tissue-engineered cartilages by seeding rabbit chondrocytes and compared bioactivity and biocompatibility between the scaffolds. Finally, we used biochemical and histological approaches to assess the tissue-engineered cartilages from qualitative and quantitative perspectives.

## 2. Materials and Methods

### 2.1. Preparation of ACECM and WJECM Scaffolds

#### 2.1.1. Pulverization and Decellularization of Human Umbilical Cord

With approval by the Ethics Committee of the Chinese PLA General Hospital and informed consent by the pregnant women, fresh umbilical cord tissues (from full-term pregnancy healthy mothers aged 20–30 years) were obtained from the obstetrics department. Then they were immersed in the electrolyzed oxidizing water (EOW), pH 2.5, for initial sterilization for 10 min, which was repeated three times. Umbilical cord tissues were cut open from the middle under sterile conditions and then peeled off the outer tissue and vascular tissue. The remaining adhesive tissues (Wharton's jelly) were rinsed with sterile distilled water for 5 min, and these procedures were repeated three times. They were subsequently placed in 3% H_2_O_2_ for sterilization 30 min and rinsed with sterile distilled water 30 min, which was repeated three times. The jelly was placed in a grinder, adding three volumes of sterile distilled water, and repeatedly crushed them into a homogenate at −5°C. Then, five volumes of sterile distilled water were added. The homogenate was frozen at −20°C and then thawed at room temperature. This freeze-thaw cycle was repeated four or five times. Using differential centrifugation approach, the homogenate was centrifuged in a centrifuge (Beckman Allegra X-22R, USA, F0850 rotor) for 30 min at 3,000 rpm. Then the supernatant fluid was taken from the homogenate. Then supernatant fluid centrifuged for 30 min at 5,000 rpm and separated again by gradient centrifugation for 30 min at 7,000 rpm. Then it was separated again by gradient centrifugation for 30 min at 10,000 rpm. The final supernatant was discarded and the precipitate was used as umbilical cord Wharton's jelly ECM (WJECM).

#### 2.1.2. Pulverization and Decellularization of Cartilage

Cartilage tissues were cut from the fresh porcine knee joints, which were purchased from local market and then rinsed in sterile distilled water and washed three times. The cartilage was immersed in the EOW (PH 2.5) for 10 min, and this was repeated three times. Under sterile conditions, the cartilage was cut into slices, 1 mm^3^ in size, and washed three times in sterile distilled water. They were immersed in 3% H_2_O_2_ for 40 min disinfection and rinsed with sterile distilled water four times. The cartilage particles were put into a homogenizer; sterile distilled water was added at 4°C; and it ground them repeatedly. The distilled water was mixed with the sterile homogenate. The homogenate was frozen at −20°C and thawed at room temperature three times. Then, the homogenate was subjected to gradient centrifugation at 3,000 rpm for 30 min (Beckman Allegra X-22R, USA, F0850 rotor). The supernatant was collected and centrifuged again at 5,000 rpm for 30 min. Then the supernatant was again centrifuged at 7,000 rpm for 30 min. Finally the homogenate was centrifuged at 10,000 rpm for 30 min. The supernatant was removed and collected the deposits as articular cartilage ECM (ACECM).

### 2.2. Fabrication of ACECM and WJECM Scaffolds

**Figure 1:**
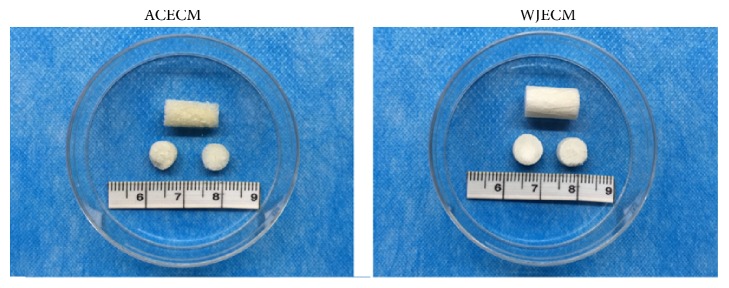
Macroscopic view presenting the surface appearance of WJECM and ACECM scaffolds.

The suspensions of ACECM or WJECM were poured into the cylindrical molds, and the scaffolds were prepared by freeze-drying method [26]. Briefly, the suspensions were first frozen at −20°C for 2 h and then frozen to −80°C for 1 h with a constant cooling rate of 1°C/min, lastly lyophilized for 48 h in a freeze-dryer (Boyikang, Beijing, China). Then scaffolds were treated with carbodiimide solution (14 m M 1-ethyl-3-(3-dimethylaminopropyl)carbodiimide hydrochloride [EDAC] and 5.5 m M N-hydroxysuccinimide [NHS]; Sigma) for 2 h at 4°C for cross-linking [4, 27]. PBS solution was used repeatedly to rinse out excess EDAC from the scaffolds. ACECM- and WJECM-derived scaffolds were prepared approximately 8 mm in diameter and 2 mm in thickness (Figure 1). All the scaffolds were sterilized by 60Co *γ* irradiation (5 mrad).

### 2.3. The Comparison of Physicochemical Properties between the Two Scaffolds

#### 2.3.1. Microstructure

Scaffolds were cut into specimens of 1 mm thick cylinders to observe the general morphology and the interior microstructures by a sharp blade. After using gold/palladium to coat the samples, scanning electron microscopy (SEM; S-4800, Hitachi, Tokyo, Japan) was used to examine the samples at 1 kV.

#### 2.3.2. Porosity

The ethanol intrusion methods were performed to measure the porosity. Firstly, add the anhydrous ethanol in a graduated tube, and record the ethanol volume as *V*_1_. Then the scaffold was placed in for 5 min, using negative-pressure degassing to exhaust gases from ethanol in the test tube until there was no air bladder overflow; we recorded the volume as *V*_2_. After taking out the scaffold, we then read the remaining volume as *V*_3_. For each scaffold group, three samples of the same size were measured separately. The porosities of the scaffolds were determined using the following formula: *E* = (*V*_3_ − *V*_2_)/(*V*_3_ − *V*_1_). Each type of scaffold was tested three times; the average value was recorded as the final result for each scaffold.

#### 2.3.3. Scaffold Swelling Properties

Using the samples' wet weight (*m*_wet_) followed by porogen leaching in triple-distilled water and drying the scaffolds in vacuo to obtain the dry weight (*m*_dry_), we calculated the water absorption capacity (%). We used the following equation to calculate the swelling ratio:

(1) $$\begin{array}{c} Swelling\;\;ratio=\frac{m_{wet}-m_{dry}}{m_{dry}}\times 100. \end{array}$$

#### 2.3.4. Compressive Properties

A miniature material tester model (minimat 2000; Rheometric Scientific, Inc., Piscataway, NJ, USA) equipped with a 20 N load cell was used to perform unconfined compression testing. Firstly, 1 mm/min cross-head speed was used until a force reaching 20 N. The elastic compressive modulus was calculated by the gradient of the first zone of linearity in the stress strain curve and the compressive strength was measured at 30–50% strain in the beginning of the plateau region. The initial compression thickness of the scaffolds (dry state) used in the above experiments was all in 2 mm.

#### 2.3.5. Comparison of Histological Characterization

The scaffolds were cut by cryosection in 8 *μ*m thickness, then fixed in acetone for 30 min at room temperature (RT), and washed with PBS. All specimens were stained with 0.1% toluidine blue, 1% safranin O, and type I and II collagen immunofluorescence staining.

### 2.4. The Construction of the Tissue-Engineered Cartilage

#### 2.4.1. Isolation and Expansion of Primary Rabbit Chondrocytes

With approval by the Ethics Committee of the Chinese PLA General Hospital, articular cartilages were harvested aseptically from the joint surfaces (shoulder joint and knee joint) of 2-month-old New Zealand White rabbits in PLA General Hospital Medical Laboratory Animal Center. Using a sterile technique, as described previously [11, 12], rabbits chondrocytes (RACs) were isolated and cultured. The cartilage was diced into fragments < 1 mm^3^. The minced cartilages were digested in regular culture medium containing 0.2% collagenase type II (Sigma, USA) and transferred to an orbital shaker overnight at 37°C. Then 0.75 × 10^6^ cells were placed in tissue culture T-75 cm^2^ flasks (Corning, USA) at ~25% confluence in culture solution (DMEM containing glucose and glutamine, 15% FBS, 120 U/ml penicillin, 120 kg/ml streptomycin, 14 mM HEPES, 0.2 mM nonessential amino acids, 0.5 mM proline, and 60 mg/L ascorbic acid) (Sigma, USA). After 1-2 weeks, cells in the T-75 flask approached 100% confluence (P0) and were trypsinized using 0.25% trypsin/EDTA (Gibco, USA) and then subcultured in T-75 flasks at a density of 1 × 10^4^ cells per cm^2^. The first subcultures were labeled as passage 1 (P1) cells, and so forth. Medium was replaced twice per week.

#### 2.4.2. Constructing of the Chondrocytes/Scaffolds Composites

After trypsin/EDTA treatment, RACs (P_2_) were collected in a centrifuge tube and washed three times with Hank's salt solution. Then RACs were seeded into the sterile ACECM and WJECM scaffolds (8 mm diameter, 2 mm thickness). The number of seeded cells was 1.0 × 10^6^ for each ACECM or WJECM scaffold disk. The chondrocytes/scaffolds composites were then placed in the 6-well culture plates and incubated in a 5% CO2 humidified atmosphere at 37°C for 30 min to allow cell adherence. Then 3 ml culture media were added to each well and cultured for 7 and 14 days, changing the culture solution every 3 days.

### 2.5. Cellular Proliferation Assay

Cell proliferation in the ACECM and WJECM scaffolds was assessed using the 3-[4,5-dimethyl(thiazol-2yl)-3,5-diphenyl]tetrazolium bromide (MTT) (Sigma) testing. Briefly, RACs (1 × 10^3^ in a 300 *μ*L suspension) were seeded in 96-well plates. After incubation with control medium and pure concentrations of extracts for 0–6 days, 20 *μ*L of MTT solution was added to each well in new culture medium. After 6 h of further incubation at 37°C, the reaction liquid was removed from each well, and 150 *μ*L of DMSO was added to dissolve the formazan crystals. A microplate reader (Beckman, Fullerton, CA) was used at 570 nm to measure the optical density. Three replicates were made per sample. The method of preparing extracts from the scaffolds was described previously [28].

### 2.6. Scanning Electron Microscopy Observation of Chondrocyte Morphology

SEM was used to observe the scaffolds' adhesion. At day 3, chondrocytes/scaffolds composites were removed from the culture medium, washed twice in PBS, and fixed for 24 h at 4°C in 2.5% glutaraldehyde (Sigma, USA). A range of 25, 50, 75, and 95% alcohol was used to dehydrate the scaffolds and after it was treated with 100% alcohol for 10 min two times. Then the scaffolds were dried at RT. After gold palladium coating, the sections were observed by SEM (S-4800, Hitachi, Tokyo, Japan).

### 2.7. Cell Viability Assessment

Cell viability was assessed using cell live/dead assay kit (Sigma, USA) after 7 and 14 days (*n* = 3 for each treatment) culture. The chondrocytes/scaffolds composites were washed twice in PBS and incubated in 5 × 10^−3^ mg/ml fluorescein diacetate (FDA) 5 min at RT in the dark. Then, the FDA was aspirated and again washed twice in PBS. The construct was incubated in another 5 × 10^−3^ mg/ml propidium iodide (PI) for 5 min at RT in the dark. PI was removed, and the construct was washed twice in PBS and examined by confocal microscopy (Olympus IX 81, Japan).

### 2.8. Biochemical Analyses

#### 2.8.1. The DNA Quantification

The DNA content in each chondrocytes/scaffolds composites was measured by Quant-iT™ PicoGreen® dsDNA assay (Invitrogen, USA) after 7 and 14 days culture, according to the manufacturer's instructions. DNA concentrations per scaffold were calculated according to the DNA standard curve used the spectrofluorometer (BioTek, Winooski, USA). Three replicates were made per sample.

#### 2.8.2. The sGAG Quantification

The sGAG content each chondrocytes/scaffolds composites was measured by Tissue GAG Total Content DMMB Colorimetry Kit (GenMed Scientifics Inc., USA) after 7 and 14 days culture, according to the manufacturer's instructions. The total sGAG concentrations per scaffold were calculated according to the sGAG standard curve. Similarly, the sGAG content in various scaffolds without seeded chondrocytes was also detected. And the pure sGAG concentrations secreted by the pure chondrocytes/scaffolds composites were calculated by total sGAG concentrations of pure chondrocytes/scaffolds composites take out the sGAG content in pure scaffold, respectively. Three replicates were made per sample.

#### 2.8.3. The Hydroxyproline Quantification

The collagen content each chondrocytes/scaffolds composites was measured by Hydroxyproline Kit (Nanjing Jiancheng Bioengineering Institute, China) after 7 and 14 days of culture, according to the manufacturer's instructions. The total hydroxyproline concentrations per scaffold were calculated according to the standard curve. Similarly, the hydroxyproline content in various scaffolds without seeded chondrocytes was also detected. And the pure hydroxyproline concentrations secreted by the pure chondrocytes/scaffolds composites were calculated by total hydroxyproline concentrations of pure chondrocytes/scaffolds composites take out the hydroxyproline content in pure scaffold, respectively. Three replicates were made per sample.

### 2.9. RNA Extraction and Quantitative PCR

RNA was extracted from chondrocytes/scaffolds composites by adding TRIzol (Life Technologies, USA). The cDNA was reverse-transcribed using ReverTra Ace® qPCR RT Master Mix (Toyobo, Japan). 1 *μ*l cDNA were amplified in a 20 *μ*l PCR mixture including Platinum SYBR Green Realtime PCR Master Mix-Plus (Toyobo, Japan) and gene-specific primers (Parkson Beijing) (Table 1) in accordance with the manufacturer's instructions. The reaction comprised an initial denaturation for 95°C for 2 min, 40 cycles with denaturing at 95°C for 15 s, and annealing and extension at 55°C for 15 s in each cycle, performed using a StepOne Real-Time PCR System (Applied Biosystems, USA).

Primer sequences were designed based on the published gene sequences (NCBI and PubMed). GAPDH was chosen as an endogenous control for the study. The relative gene expression profiles of various samples were normalized to the corresponding GAPDH and analyzed using the 2^−ΔΔCT^ approach. Three replicates were made per sample.

### 2.10. Histological and Immunofluorescence Analyses

The chondrocytes/scaffolds composites after 7 and 14 days in culture were cut by cryosection in 8 *μ*m thickness, then fixed in acetone for 30 min at room temperature (RT), and washed with PBS solution. On the one hand, some specimens were stained with hematoxylin and eosin (H&E), 0.1% toluidine blue, and 1% safranin O; on the other hand, the other samples were assessed by type I collagen (Abgent, USA), type II collagen (Abgent, USA), and aggrecan (Novus, USA) immunofluorescence staining. Secondary antibodies (Jackson, USA) and an Immunofluorescence Staining Kit (Vector Laboratories, UK) were used in accordance with the manufacturer's protocols. Negative controls were processed in parallel with no primary antibody.

### 2.11. Statistical Analysis

Quantitative results are shown as means ± standard deviations. Statistical analyses were carried out using one-way ANOVA. *p* values < 0.05 were considered to indicate statistical significance. SPSS software ver. 17.0 (SPSS Inc., Chicago, IL, USA) was used for the analyses.

## 3. Results

### 3.1. Physicochemical Properties of ACECM and WJECM Scaffolds

**Figure 2:**
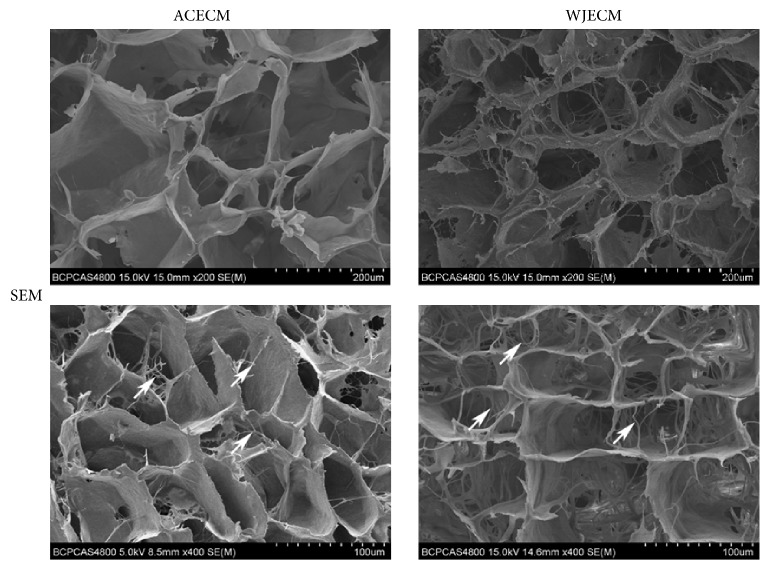
SEM image showing the surface structures of WJECM and ACECM scaffolds. The white arrow indicates that nanofibrous ACECM and WJECM in the both various scaffolds.

**Figure 3:**
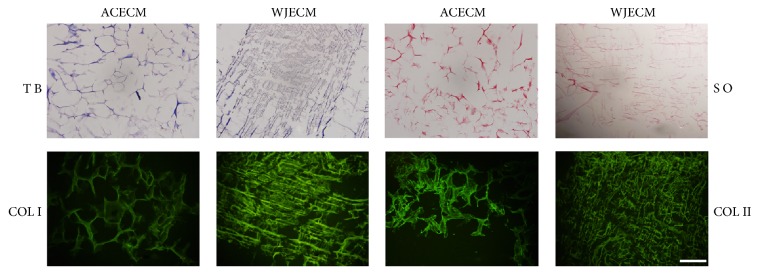
Component identification in WJECM and ACECM scaffolds. Toluidine blue staining and safranin O staining were positive; type I and type II collagen immunofluorescence showed positive staining (scale bar = 200 *μ*m).

The scaffolds showed uniformly interconnected pore structures, as observed by SEM (Figure 2). It could be seen that there are nanofibrous ACECM and WJECM in the both various scaffolds. The ACECM scaffold had a mean pore size of 193.6 ± 62.1 *μ*m and mean porosity of 75.7 ± 10.5% (Table 2). For the WJECM scaffold, the mean pore diameter was 127.4 ± 42.2 *μ*m and the average porosity was 84.6 ± 3.2% (Table 2). The mean pore size and porosity did not differ significantly between the scaffolds. However, the water uptake ratio of the ACECM scaffold (31.1 ± 5.5) was much higher than that of the WJECM scaffold (16.7 ± 2.3). Regarding the compressive modulus, that of the WJECM scaffold (379.2 ± 28.5 Pa) was higher than that of the ACECM scaffold (297.9 ± 17.9 Pa). Histological staining was used to compare the biochemical composition between the ACECM and WJECM scaffolds. Safranin O, toluidine blue, and type I and type II collagen immunofluorescent staining confirmed that the two different scaffolds both contained the same components of sGAG and collagens (Figure 3).

### 3.2. Cell Proliferation and Adhesion in ACECM and WJECM Scaffolds

**Figure 4:**
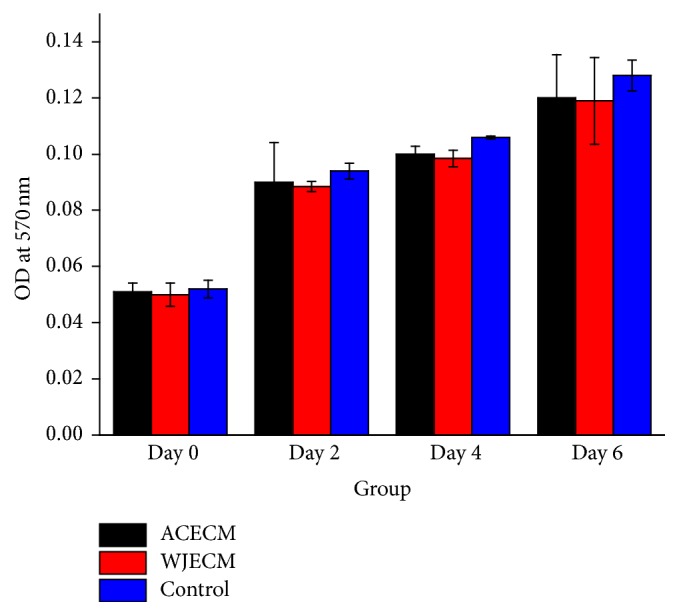
MTT assay showing the proliferation of chondrocytes cultured in WJECM and ACECM scaffolds in comparison with the control group after 0, 2, 4, and 6 days of culture (*n* = 6). Both WJECM and ACECM scaffolds showed favorable cell proliferation rates.

**Figure 5:**
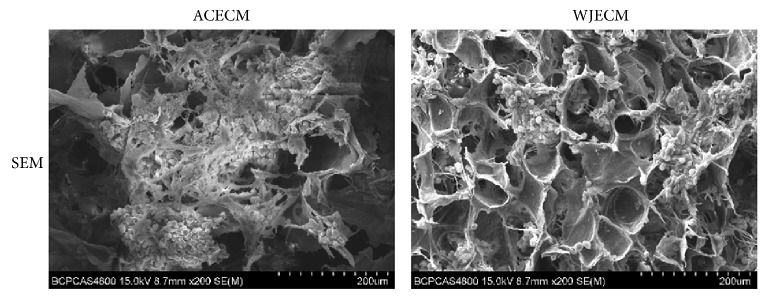
SEM analysis showing chondrocyte adhesion on both WJECM and ACECM scaffolds after 3 days in culture.

Cell proliferation in the ACECM and WJECM scaffolds was assessed using the MTT quantitative assay after 0, 2, 4, and 6 days in culture (Figure 4). The cell number increased in both scaffolds and the control group with time. The cell proliferation capacity in the ACECM and WJECM scaffolds did not show significant differences at 0, 2, 4, or 6 days (*p* > 0.05). Cell morphology and adhesion in the ACECM and WJECM scaffolds were observed from SEM images after 3 days in culture (Figure 5). The cells in both groups maintained their spherical morphology well and gathered into clusters, showing some ECM secretion. The cell numbers adhering to the scaffolds did not show a significant difference.

### 3.3. Cell Viability in ACECM and WJECM Scaffolds

**Figure 6:**
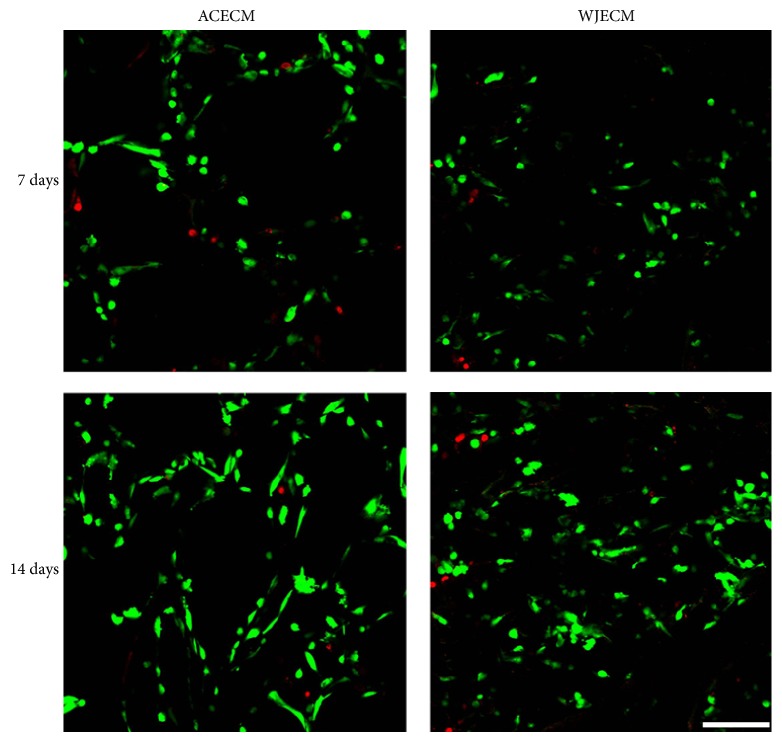
Live/dead cell staining of chondrocytes cultured on WJECM and ACECM scaffolds 7 and 14 days after seeding. The live cell number increased from days 7 to 14 in both groups. Regarding live cell numbers, the two scaffolds showed no significant difference at day 7 and day 14 (scale bar = 200 *μ*m).

Cell viability in the ACECM and WJECM scaffolds was assessed using the live/dead assay after 7 and 14 days in culture (Figure 6). Both groups showed live cells at days 7 and 14 (green); moreover, the live cell number increased from days 7 to 14. These results were consistent with the MTT assay. Regarding live cell numbers, the groups showed no significant difference as day 7 and day 14.

### 3.4. Biochemical Assessment

#### 3.4.1. DNA Assessment

**Figure 7:**
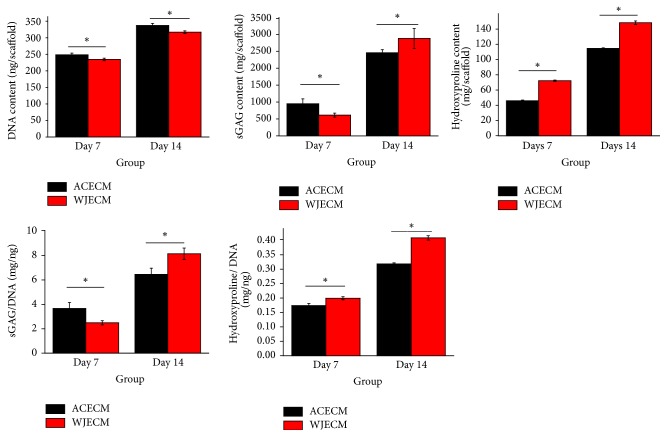
DNA, sGAG, and collagen production by chondrocytes cultured on WJECM and ACECM scaffolds 7 and 14 days after seeding. Data represent the mean ± SD of three independent experiments. ^*∗*^*p* < 0.05.

The DNA contents of chondrocytes on the scaffolds were quantified at 7 and 14 days after seeding. The DNA content increased in both ACECM and WJECM scaffolds with time; it was slightly higher in the ACECM group than in the WJECM group at 7 and 14 days. This trend was consistent with the MTT assessment (Figure 7).

#### 3.4.2. sGAG Assessment

sGAG production in chondrocytes in the scaffold was higher in the ACECM group than in the WJECM group at 7 days. sGAG production increased at 14 days in both scaffolds, while it was higher in the WJECM group than in the ACECM group. Using sGAG content normalized to the corresponding DNA to assess sGAG production, the sGAG/DNA content in the WJECM group was higher than that in the ACECM group at 7 and 14 days (Figure 7).

#### 3.4.3. Total Collagen Assessment

The total collagen production of chondrocytes was represented by hydroxyproline. The total hydroxyproline production in the scaffolds was higher in the WJECM group than in the ACECM group at 7 and 14 days. The total hydroxyproline production increased from 7 to 14 days in both scaffolds. Using total hydroxyproline content normalized to corresponding DNA to assess collagen secretion, the hydroxyproline/DNA content in the WJECM group showed the same trend as pure hydroxyproline production at 7 and 14 days (Figure 7).

### 3.5. RT-qPCR Assessment

**Figure 8:**
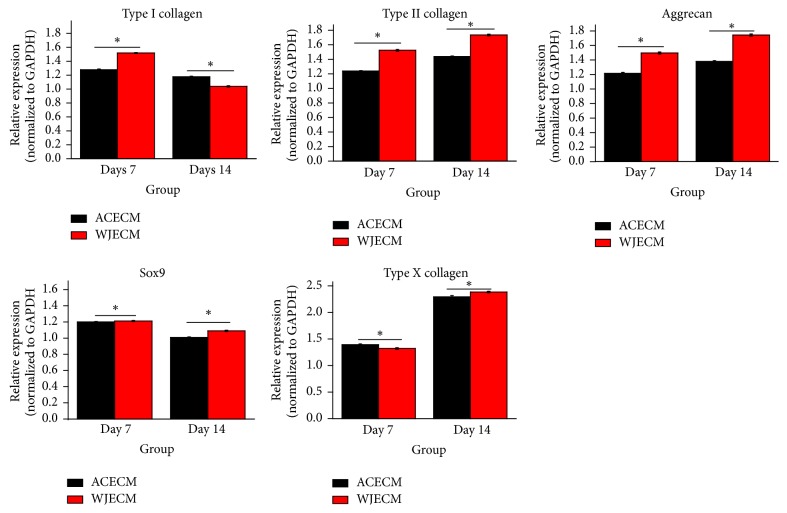
RT-qPCR gene expression analyses of chondrocytes cultured on WJECM and ACECM scaffolds 7 and 14 days after seeding. All data were normalized to the corresponding GAPDH value at 7 and 14 days after seeding (Δ*C*_*T*_) and further normalized to the* ΔC*_*T*_ value of the target gene in the control (2^−ΔΔCT^). Results are reported as mean fold change ± SD from three independent experiments. ^*∗*^*p* < 0.05.

For further assessment of the effects of the scaffolds on the gene expression characteristics of the chondrocytes, mRNA levels of cartilage ECM components (collagen I, collagen II, aggrecan, and collagen X) and a transcription factor (Sox-9) were detected by RT-qPCR. Regarding chondrogenic gene expression markers (collagen II and aggrecan), they showed increasing trends in both scaffolds, and collagen II and aggrecan gene expression levels were higher in the WJECM group than in the ACECM group at 7 and 14 days. Sox-9 gene expression was higher in the WJECM group than in the ACECM group at 7 days and decreased at 14 days, although it was higher in the WJECM group than in the ACECM group at 14 days. Collagen I expression was higher in the WJECM group than in the ACECM group at 7 days and decreased at 14 days, although it was lower in the WJECM group than in the ACECM group at 14 days. Collagen X gene expression showed an increasing trend in both groups from 7 to 14 days. It was lower in the WJECM group than in the ACECM group at 7 days; however, it increased more in the WJECM group than in the ACECM group at 14 days (Figure 8).

### 3.6. Histology and Immunofluorescent Staining

**Figure 9:**
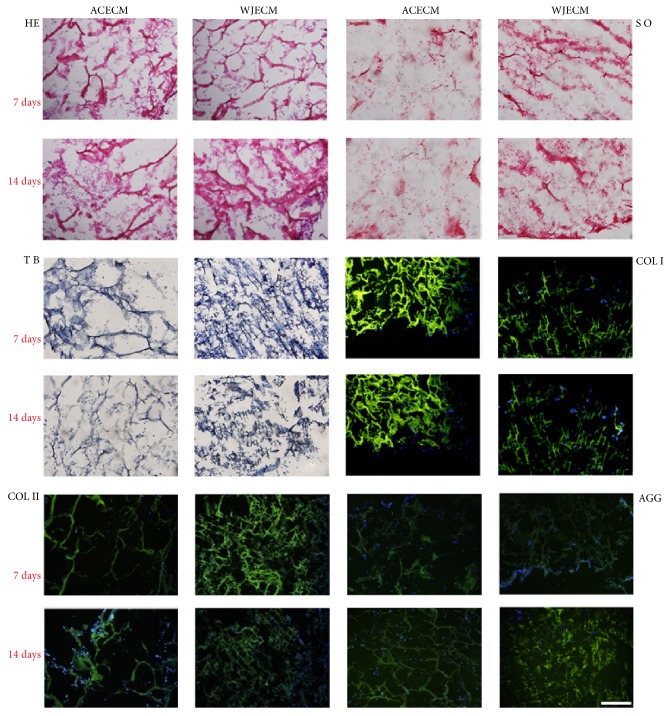
Histological and immunofluorescent staining of chondrocytes cultured on WJECM and ACECM scaffolds 7 and 14 days after seeding (scale bar = 200 *μ*m). H&E staining showed that the cells were distributed evenly and secreted ECM in both of the scaffolds. Safranin O and toluidine blue staining showed that chondrocytes in the WJECM scaffold showed more intense staining than those in the ACECM scaffold at 7 or 14 days. At 7 and 14 days, both scaffolds were positive for collagen I, collagen II, and aggrecan.

Histology and immunostaining were performed to assess the cells and specific matrix distributions in the scaffolds. H&E staining confirmed that the cells were distributed evenly and secreted some ECM in the two scaffolds at 7 and 14 days. Safranin O and toluidine blue staining demonstrated that chondrocytes in the WJECM scaffold showed more intense staining than in the ACECM scaffold at 7 and 14 days, consistent with the sGAG assessment. At 7 and 14 days, both scaffolds showed positive staining for collagen I, collagen II, and aggrecan; however, there was no significant difference (Figure 9).

## 4. Discussion

Researchers are currently focusing on cartilage tissue engineering because of the increasing incidence of articular cartilage injuries [29]. Some progress has been made in terms of cartilage regeneration [30–32]. The scaffold plays an important role in providing a suitable growth environment for seeding cells and directing new tissue regeneration [33, 34]. Decellularized articular cartilage ECM (ACECM) was thought to be the ideal biomaterial for tissue-engineered cartilage due to its biomimetic ECM composition and proteins [21]. Decellularized Wharton's jelly ECM (WJECM) is not only similar to ACECM but also possesses some chondrogenic growth factors [22–24]. Our hypothesis was that WJECM would be a good tissue-engineered cartilage biomaterial, in comparison with ACECM.

We used a waterproof pulverization and differential centrifugation approach to obtain two different decellularized ECMs and prepared two scaffolds with the constant cooling freeze-drying method. On the one hand, the pulverization and centrifugation approach was beneficial to separate and obtain the ECM suspensions from the Wharton's jelly or cartilage; on the other hand, the constant cooling technique can be in favor of fabricating a uniform pore structure by forming uniform temperature distribution during the freezing process as O'Brien and colleagues have confirmed [26]. SEM images showed that both ACECM and WJECM scaffolds displayed a uniform porous sponge structure, which may be beneficial for nutrient transportation and metabolic waste exclusion. The mean pore sizes and porosities of the two scaffolds were comparable and showed no statistically significant difference. Both ACECM and WJECM scaffolds were hydrophilic; however, the swelling ratio of ACECM was larger than that of WJECM. We speculate that different ECM component may lead to various swelling ratio; therefore ACECM scaffold may have more absorbent materials than that of the WJECM scaffold. The good hydrophilicity may enhance the biocompatibility of both scaffolds.

It is especially important for scaffolds with good biomechanical properties to withstand normal human compression forces when performing cartilage repair surgery. Thus, it is important to prepare cartilage scaffolds with favorable mechanical strength. The compressive modulus of the WJECM scaffold was higher than that of the ACECM scaffold. We concluded that the WJECM scaffold could resist more powerful compressive stimulation than the ACECM scaffold. Histological staining further confirmed that the WJECM and ACECM scaffolds contained similar components of sGAG, collagen I, and collagen II, mimicking native cartilage ECM. The WJECM scaffold may provide a biomimetic environment for seed cell proliferation and redifferentiation.

The biocompatibility of the scaffold plays a key role in tissue regeneration and functional reconstruction. The results of the SEM assessment showed that both ACECM and WJECM scaffolds had good cell adherence at 3 days after seeding. We suggest that the favorable attachment may be associated with the collagen components in both scaffolds because collagen enhances cellular adhesion. The MTT assay and cell viability test demonstrated that the cells adhered on both scaffolds and proliferated rapidly with time. The increased chondrocyte number and viability on both scaffolds suggested that both scaffolds had good cell affinity and could mimic the native cartilage ECM microenvironment for cell adhesion and proliferation.

Both ACECM and WJECM scaffolds were rich in sGAG and collagens; moreover, the WJECM contained more growth factors than the ACECM, such as IGF-I and TGF-*β*, which would be highly conducive to seed cell chondrogenesis [24]. This is an important point because favorable scaffolds can improve the formation of tissue-engineered cartilage. RT-qPCR confirmed that the WJECM groups showed more collagen II and aggrecan mRNA expression. The upregulation of collagen II may be related to the higher Sox-9 mRNA expression in the WJECM group because Sox-9 can upregulate collagen II expression by specifically combining with an enhancer element [35, 36]. Moreover, collagen and sGAG quantitative tests further confirmed the RT-qPCR results, demonstrating more collagen and sGAG production in the WJECM group. Safranin O and toluidine blue staining was more intense in the WJECM group than in the ACECM group. However, the immunofluorescent staining showed no significant difference, although this may have been due to insufficient culture time.

The DNA contents in the groups reflect cell numbers in the scaffolds; the results were consistent with the MTT results, showing more cell proliferation in the ACECM group than in the WJECM group. We suggest that the growth factors in the WJECM scaffold may enhance cellular chondrogenesis more than proliferation. Collagen I gene expression was initially upregulated and then decreased in both groups, while expression in the WJECM group was ultimately lower than in the ACECM group. This was consistent with the cellular chondrogenesis results. Collagen X gene expression is associated with osteogenic differentiation [37, 38]. Gene expression of collagen X was upregulated in both groups; it was ultimately higher in the WJECM group than in the ACECM group.

This investigation has some limitations. First, it may be necessary to extend the culture times to induce chondrocyte chondrogenesis because we did not obtain lacuna structures in the cells. Second, we may need to examine different components specifically between WJECM and ACECM, especially in terms of growth factors, to explain further the chondrogenesis mechanism in the two ECMs. Finally, although WJECM can upregulate the expression of some chondrogenic markers, to avoid osteogenic differentiation, we may attempt to set up chondrogenic culture conditions that decrease collagen X expression.

## 5. Conclusions

In this study, we prepared WJECM scaffolds using a waterproof pulverization and differential centrifugation approach with the freeze-drying method. The WJECM scaffolds showed favorable bioactivity and biocompatibility characteristics. In conclusion, WJECM scaffolds may be valuable for applications in cartilage tissue engineering.

## Figures and Tables

**Table 1:** Primer sequences of target genes used for RT-qPCR.

Target gene	Primer sequence	NCBI accession number
GAPDH	F: 5′-CAAGAAGGTGGTGAAGCAGG-3′ R: 5′-CACTGTTGAAGTCGCAGGAG-3′	NM_001082253.1
Collagen I (col1a2)	F: 5′-GCCACCTGCCAGTCTTTACA-3′ R: 5′-CCATCATCACCATCTCTGCCT-3′	NM_001195668.1
Collagen II (col2a1)	F: 5′-CACGCTCAAGTCCCTCAACA-3′ R: 5′-TCTATCCAGTAGTCACCGCTCT-3′	XM_002723438.1
Collagen X (col10a1)	F: 5′-CCACCAGGACAAGCAGTCAT-3′ R: 5′-CACTAACAAGAGGCATCCCG-3′	XM_002714724.1
Sox-9	F: 5′-GCGGAGGAAGTCGGTGAAGAAT-3′ R: 5′-AAGATGGCGTTGGGCGAGAT-3′	XM_002719499
Aggrecan	F: 5′-GGAGGAGCAGGAGTTTGTCAA-3′ R: 5′-TGTCCATCCGACCAGCGAAA-3′	XM_002723376.1

Sox-9, (sex determining region Y)-box 9.

**Table 2:** The physicochemical properties between ACECM and WJECM scaffold. Results are reported as mean fold change ± SD from three independent experiments. ^*∗*^*p* < 0.05.

Characteristic	ACECM scaffolds	WJECM scaffolds
Mean pore size (*μ*m)	193.6 ± 62.1	127.4 ± 42.2
Porosity (%)	75.7 ± 10.5	84.6 ± 3.2
Water swelling ratio	31.1 ± 5.5	16.7 ± 2.3^*∗*^
Compressive modulus (Pa)	297.9 ± 17.9	379.2 ± 28.5^*∗*^
